# Parenthood and Race Culture

**Published:** 1909-12

**Authors:** 


**Parenthood and Race Culture.** An Outline of Eugenics. By **Caleb Williams Saleby,** M.D., Ch.B., E.R.I., Edin., Fellow of the Obstetrical Society of Edinburgh. New York: Moffat, Yard & Co. (Cloth, $2.50.)

This is a useful and interesting book, the nature of which is well indicated by its title. The author states it is the first comprehensive work on the subject, which he is pleased to call Eugenics. He discusses in a thoroughly broadminded and liberal way, the general principles of race culture, defines the limits of education, recognises the importance of heredity, and seeks to show how race culture may be practised even in the present state of social sentiment. He discourages parenthood on the part of the insane, chronic inebriates and feeble-minded, in all of which every sensible person will agree with him.

